# Notch Signaling in Kidney Development, Maintenance, and Disease

**DOI:** 10.3390/biom9110692

**Published:** 2019-11-04

**Authors:** Malini Mukherjee, Eric Fogarty, Madhusudhana Janga, Kameswaran Surendran

**Affiliations:** 1 Pediatrics and Rare Diseases Group, Sanford Research, 2301 East 60th Street North, Sioux Falls, SD 57104, USA; malini.mukherjee@sanfordhealth.org (M.M.); Madhusudhana.Janga@sanfordhealth.org (M.J.); 2 Division of Basic Biomedical Sciences, Sanford School of Medicine, University of South Dakota, Vermillion, SD 57069, USA; Eric.Fogarty@sanfordhealth.org; 3 Department of Pediatrics, Sanford School of Medicine, University of South Dakota, Sioux Falls, SD 57105, USA

**Keywords:** Alagille syndrome, Notch, congenital anomalies of the kidney, nephrogenesis, cell fate selection

## Abstract

Kidney development involves formation of nephrons intricately aligned with the vasculature and connected to a branched network of collecting ducts. Notch signaling plays multiple roles during kidney development involving the formation of nephrons composed of diverse epithelial cell types arranged into tubular segments, all the while maintaining a nephron progenitor niche. Here, we review the roles of Notch signaling identified from rodent kidney development and injury studies, while discussing human kidney diseases associated with aberrant Notch signaling. We also review Notch signaling requirement in maintenance of mature kidney epithelial cell states and speculate that Notch activity regulation mediates certain renal physiologic adaptations.

## 1. Introduction

The Notch signaling pathway is an evolutionarily conserved signaling pathway that has critical functions in embryonic development, tissue homeostasis, and disease. Unique features of the pathway include ligand and receptor expression in neighboring cells and a series of proteolytic cleavages of the receptor that lead to the expression of Notch target genes. After exit from the endoplasmic reticulum, the Notch receptors undergo processing in the Golgi where they are cleaved by furin-like convertases, resulting in a bipartite receptor that translocates to the plasma membrane [1]. Notch receptor activation occurs when ligands on neighboring cells bind to the receptor extracellular domain, and ligand endocytosis opens up the negative regulatory region within the extracellular domain [2] to allow for ADAM metalloproteases to cleave the Notch receptor [3]. This then triggers the gamma secretase protein complex to mediate cleavage within the Notch transmembrane domain [4,5,6]. The released Notch intracellular domain (NICD) translocates into the nucleus and activates Notch target genes (notably members of the Hes and Hey family) by forming an activator complex with CSL [CBF1/Su(H)/Lag-1] and the co-activator Mastermind-like and additional transcription activation complex [7,8].

In mammals, there are four Notch receptors (Notch1–Notch4) and five ligands [Delta-like (Dll1, Dll3, and Dll4) and Jagged (Jag1 and Jag2)]. These basic components, along with their downstream mediators orchestrate gene regulation in almost all tissues of multicellular organisms. Research conducted for over a century has contributed to our understanding of the mechanism of Notch regulated gene expression in a context dependent manner (reviewed in [9,10], focusing on mechanism of finer aspects of Notch regulation). Notch signaling has a role in the development of multiple organs including the liver [11,12], brain [13], bone [14], in addition to playing a role in malignancies of multiple organs [15]. Considering that several human kidney diseases have been associated with Notch signaling, including Alagille syndrome, Hadju-Cheney syndrome, congenital anomalies of the kidney and urinary tract (CAKUT), and diabetic nephropathy, here we review the functions of Notch signaling in kidney development and maintenance in order to gain insights into Notch-associated kidney diseases.

## 2. The Myriad Functions of Notch Signaling during Kidney Development

### 2.1. Overview of Kidney Development

Notch signaling plays multiple roles during the development of the permanent mammalian kidney, the metanephros, which is initiated when glial derived neurotrophic factor (GDNF) signals from the metanephric mesenchyme to trigger ureteric budding from an epithelial tubular structure referred to as the Wolffian duct. The tip cells of the ureteric bud (UB) express high levels of Ret and GFRα1 receptors that are activated by GDNF [16], and facilitate the invasion and repeated branching of the UB within the metanephric mesenchyme. The UB tip cells are progenitors of the collecting ducts as they proliferate to give rise to both tip cells and to the cells in the trunk of the UB [17]. Interestingly, Ret signaling, which activates Etv4 and Etv5 in the UB tips [18], is required for cells to remain in the UB tip as Ret or Etv4 and Etv5 mutant cells tend to be excluded from the UB tips [17,19,20]. Extensive branching of the UB gives rise to the collecting duct system [21], while the Six2 expressing metanephric mesenchymal cells that condense around the branching end of UB give rise to nephrons [22,23]. This Six2 positive population, also referred to as the cap mesenchyme, consists of the nephron progenitor cells (NPC) from which all nephrons, the basic structural and functional units of the kidney, originate. Six2 expression is essential for maintenance of the NPC state, and cessation of Six2 expression marks the end of new nephron formation [22,24]. As discussed below, both GDNF-Ret signaling and Six2 expression can be modulated by Notch signaling to alter UB branching as well as maintenance of the NPC state [25,26].

Nephron formation occurs through a series of morphological changes that transform groups of cap mesenchymal cells that periodically exit the progenitor pool, into a tubular structure referred to as a nephron. Each nephron arises from an aggregate of cap mesenchymal cells, referred to as a pre-tubular aggregate (PTA) that forms below the UB tip. Mesenchymal aggregates epithelialize to form renal vesicles (RV) [27,28], which then become comma-shaped bodies that undergo further differentiation into S-shaped bodies. The vasculature invades at one end of the S-Shaped body to give rise to the glomerulus while the other end becomes the connecting segment that fuses with the collecting duct system [27,28]. Nephron formation initiates at the branching UB tips repeatedly from embryonic day 11.5 to a few days after birth (P3) in mice [29,30,31]. During the morphological conversion of mesenchymal aggregates into nephrons, the cells compartmentalize into nephron segments and differentiate to diverse cell types that compose each nephron. Both the expression pattern of Notch signaling pathway components as well as functional studies reveal roles for Notch signaling in nephron segmentation and differentiation [32,33,34,35]. While the collecting duct structure is more simplified compared to the nephron, the collecting ducts are also composed of distinct segments, with each segment composed of at least two distinct cell types. Much like in the nephrons, Notch signaling plays a critical role in patterning the cell types of the kidney collecting ducts [36,37].

### 2.2. Expression Pattern of Notch Pathway Components during Kidney Development

**Figure 1:**
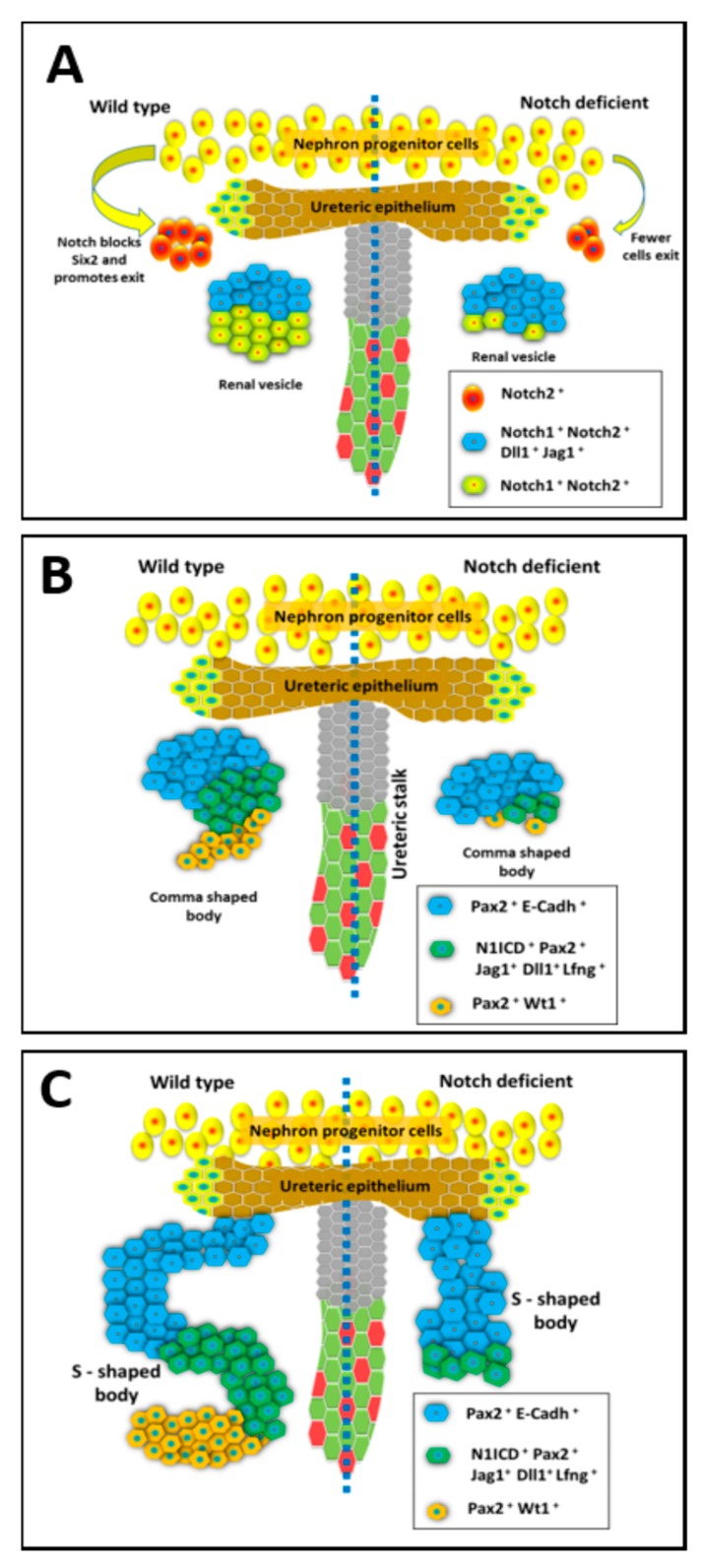
The expression and function of Notch during the early stages of Nephrogenesis. The different stages of mammalian renal nephron development with normal development (on left side) compared to kidney development under deficient Notch signaling conditions (on right side). (**A**) The nephron progenitor state is maintained through Six2 expression which is normally repressed by Notch signaling to allow cells to begin differentiation within pre-tubular aggregates that become renal vesicles (RV). The RV have distinct segments with Notch ligands having a restricted expression in the distal compartment close to the ureteric bud UB tip. Notch deficiency within the nephron progenitors results in abnormal patterning of the RV. (**B**) Diagram of the developing nephron at the comma shaped stage. The renal vesicle undergoes further morphological changes from spherical body to comma shaped body. Notch deficiency results in decreased proliferation of Pax2+; Jag1+ cells. (**C**) Diagram of the developing nephron at the S-shaped stage, which occurs after the comma-shaped stage. The S-shaped body contains distinct proximal, medial, and distal domains. Notch components are expressed differentially in this structure and function to further define the nephron segments. Inactivation of Notch alters nephron segmentation, leads to loss of proximal and medial segments, and abnormal nephron structure.

The Notch pathway receptors and ligands have unique expression patterns within the developing kidney (Figure 1) [35,38]. The Notch ligand, Jagged1 (Jag1) is expressed in the renal vesicles (RV), but only in the cells closest to the UB tip in the developing mouse and human kidneys [39]. Similarly, the Notch ligand Delta-like 1 (Dll1) is expressed only within the cells closest to the UB tip of the mouse kidney late PTA/early RV structures [40]. Within the S-shaped body (SSB) both Jag1 and Dll1 are expressed in the cells that make up the middle portion of the SSB as visualized in Figure 1 [41]. The Notch1 and Notch2 receptors are expressed in all cells of nephrogenic structures from PTA/RV stage onwards, while Notch2 expression is also detectable in NPCs. Interestingly, lunatic-fringe (Lfng), which can post-translationally modify Notch receptors to modulate their responsiveness to Jag1 and Dll1, is expressed prominently in the middle portion of SSB along with Dll1 and Jag1 (Figure 1) [41]. Within the collecting duct lineage Notch1, Notch2, and Notch3 expression have been observed along with Jag1, Dll1, and Hes1 [26,35,41,42,43].

### 2.3. Notch Signaling Represses Six2 to Promote Exit from Nephron Progenitor Niche

The recurring process of nephron formation at UB tips relies on balancing the number of nephron progenitor cells (NPCs) exiting the stem-like state to begin differentiation into mature nephron cell types with sufficient number of NPCs self-renewing and remaining in reserve within progenitor niche for subsequent rounds of nephron development. Notch signaling regulates this balancing act by repressing Six2 expression to allow for exit from the progenitor niche. As mentioned above, early cessation of Six2 expression causes the entire progenitor population to prematurely differentiate resulting in fewer nephrons per kidney [44]. Mouse kidney development studies reveal a critical role for Notch signaling in regulating the exit of Six2 positive cells from the NPC population. Inactivation of Notch components (*RBPJ*, or *Notch1* and *Notch2*) sustain Six2 expression within the developing mouse kidney [25] much after its expression should have been downregulated (Table 1). Consistent with Notch signaling regulating the NPC self-renewal versus differentiation, forced expression of ectopic NICD1 (activated Notch1) or NICD2 within the Six2-expressing cells causes precocious differentiation of the NPCs [40,45]. The finding that Notch signaling regulates renewal versus differentiation of NPCs may have utility in culturing NPCs as a source of rebuilding kidneys and to understanding mechanisms regulating nephron endowment [46]. Transient inhibition of Notch signaling within the NPC may prolong the self-renewing state as seen through increased Six2 staining [25], and hence understanding how Notch signaling is activated within the Six2 population is of great importance.

### 2.4. Notch Signaling Mediates Nephron Segmentation during Conversion of RV to SSB

Notch signaling continues to a play a role in nephron development following down-regulation of Six2 and exit from the NPC self-renewal state. Utilizing different Cre drivers to inactivate *Notch1* and/or *Notch2* within the nephrogenic lineage of the developing kidney reveals morphological defects in the conversion of RV into SSBs [40,42,51]. Early experiments involving inactivation of *Notch2* using Cre driven by *Pax3* promoter indicated a role for Notch2 signaling in the acquisition of proximal nephron cell fates including that of proximal tubule and podocytes [40], similar to what was observed with pharmacologic inhibition of Notch signaling in explant mouse developing kidney cultures using Difluorophenacetyl)-l-alanyl]-S-phenylglycine t-butyl ester (DAPT) to inhibit Notch signaling (Table 1) [33]. The Pax3-Cre system targets the metanephric mesenchyme even prior to the formation of cap condensates and hence using this system to genetically inactivate *Notch2* reveals the most severe renal defects caused by the loss of Notch2. Careful analysis of the *Pax3-Cre;Notch2f/f* mice reveals a requirement for Notch2 in establishing/maintaining distinct compartments of gene expression from the RV stage onwards. Polarized expression of Lhx1 in the top (distal) compartment of the RV closer to the UB occurs in *Pax3-Cre;Notch2f/f* kidneys just like in wild-type kidneys. However, the segment specific expression of Lhx1 is not maintained in the absence of Notch2 and the establishment of distinct nephron segments is defective from the RV stage onwards. Interestingly, Six2-Cre which drives Cre expression in the NPCs also reveals the requirement of Notch signaling in the establishment of the proximodistal axis within the RV and hence nephron segmentation, but only when both *Notch1* and *Notch2* are inactivated [51]. The RVs of *Six2-Cre;Notch1f/f;Notch2f/f* do not have Lhx1 expression restricted to the distal compartment, indicating a failure of the nephron segmentation process. The early Pax2-cre mediated inactivation of *Notch1* does not alter nephron formation in kidney explant cultures, while inactivation of *RBPJ* does inhibit proximal tubule and podocyte development [40]. A role for Notch1 in nephrogenesis is revealed when it is inactivated in a sensitized Notch signaling background created by inactivation of *Notch2* using Six2-Cre line. While most *Six2-Cre;Notch2f/f* mice develop normal sized kidneys with numerous proximal tubules, the additional inactivation of one allele of *Notch1 in Six2-Cre;Notch2f/f;Notch1f/+* mice causes most of these to develop small kidneys with reduced number of nephrons, due to defective nephron segmentation during the conversion of RV into S-shaped bodies (Table 1) [42]. These experiments show that Notch2 plays a critical role early in nephrogenesis, and although Notch1 functions at the same time it cannot compensate for the loss of Notch2 when *Notch2* is inactivated early during kidney development, but can do so when *Notch2* inactivation is initiated from the NPC stage onwards. A critical factor to consider in interpreting the outcome when conditionally inactivating genes is that wild-type mRNA and protein may be made prior to conditional genetic inactivation of a gene and that mRNA/protein may have a long half-life. The different phenotypes observed in the *Six2-Cre;Notch2f/f* versus *Pax3-Cre;Notch2 f/f* mouse kidneys is likely due to the presence of sufficient wild-type Notch2 protein during the RV to S-Shaped body conversion in the *Six2-Cre;Notch2f/f* but not in the *Pax3-Cre;Notch2 f/f* kidneys. Additionally, many studies using renal conditional inactivation of Notch signaling components have relied on Cre-mediated recombination of LoxP sites at Cre reporter loci (Rosa +/EYFP) to identify cells with successful inactivation genes in the Notch signaling pathway. This has been justified due to difficulties in staining for Notch signaling components such as Notch1 and Notch2 receptors. However, reliance on Cre reporters is potentially problematic as reporter lines only indicate that Cre-mediated recombination occurred at the reporter locus and does not always accurately inform on the status of LoxP recombination at a separate locus containing the target floxed alleles [53].

Interestingly, delaying Cre expression to the pre-tubular aggregate stage just prior to RV formation, using the Wnt4-Cre to inactivate both *Notch1* and *Notch2* along with a Rosa+/EYFP Cre reporter also results in defective nephrogenesis [51]. Mutant kidneys with *Notch1* and *Notch2* inactivation showed a deficiency in the expression of mature markers (*Wt1*, *LTL*, *Slc12a1*, or *Slc12a3*) of all nephron segments. The delayed inactivation of *Notch1* and *Notch2* using the Wnt4-Cre allowed for the initial segmentation of the RV along proximodistal axis but reduced the expression of these early stage nephron segment specific markers *Lhx1* at later stages resulting in the failure to form normal SSBs and nephrons [51]. Downregulation of Notch signaling within the developing nephron leads to a decrease in both Hnf1b and Lhx1 indicating that Notch functions in part through these factors to allow for proper nephron development [51]. The expression of *Lhx1* in the distal RV compartment and its requirement for *Dll1* expression in distal compartment of the RV is suggestive that both Notch signaling and Lhx1 are critical for development of distinct cell types within the RV [24].

The failure to form SSBs in the *Wnt4-Cre;Notch1f/f;Notch2f/f* kidneys clearly implicates a critical requirement for Notch signaling subsequent to suppressing Six2 expression and allowing NPCs to exit the progenitor state. This later function involves the proper conversion of RVs to SSBs. At the cellular level this conversion step requires the sufficient proliferation of Jag1+ cells within the early stage nephrogenic bodies [40], and at the molecular level is likely to involve sustained expression of *Lhx1* and *Hnf1b* [51].

### 2.5. Loss of Notch Signaling Allows for the Formation of Proximal Tubular Cysts and Microadenomas

In addition to playing critical roles in the early stages of nephrogenesis, Notch signaling continues to be involved in the later stages of nephron morphogenesis. The inactivation of *Notch1* alone or *Notch2* alone within Six2 expressing cells allows for normal nephron formation but results in proximal tubular cysts (Table 1) [48]. Interestingly, inhibition of Notch signaling by expressing dominant-negative mastermind-like1 (dnMaml) from after the formation of SSB also results in proximal tubular cysts [54]. In this system, Pax8-> rtTA is used to turn on TRE-> dnMaml in a doxycycline dependent manner within the SSB and developing collecting ducts and results in collecting duct cysts in addition to proximal tubular cysts. The loss of Notch signaling results in abnormal orientation of renal epithelial cell division during nephron growth and elongation, allowing for epithelial cells to stratify and form polyp-like structures within the renal tubular cysts instead of remaining in a monolayer following cell division. The renal epithelial cells maintain apical-basal polarity with the loss of Notch signaling but have abnormally long cilia. These observations are suggestive that partial loss of Notch signaling alters renal tubule morphogenesis from after the SSB stage and allows for the formation of proximal tubular cyst [48]. Notch signaling therefore plays a very important role not only in nephron segmentation and proper maturation/exit of progenitors early on but continues to suppress aberrant cellular growth. Aged out Six2-Cre; Notch2f/f mice develop microadenomas within the cysts that resemble precursors to papillary renal cell carcinoma. Interestingly, human type I papillary renal cell carcinoma is linked with reduced Notch signaling [48]. These observations are suggestive that there are additional roles for Notch signaling after the formation of SSBs in ensuring normal proximal tubule morphogenesis.

### 2.6. Notch Signaling is Required for Patterning the Collecting Duct Cell Types

The renal collecting duct network formed by the branching UB connects all the developing nephrons to the ureter. During the growth and branching of the UB, the cells differentiate into intercalated cell types intermingled among principal cell types. The principal cell types express the water channel Aquaporin-2 and are critical for water homeostasis, while the intercalated cell types express Foxi1 and anion exchangers, and are critical for pH balance. Notch signaling plays a critical role in patterning of cell types along the collecting ducts. While ectopic expression of activated Notch1 in the developing collecting duct system promotes principal cell fate selection at the expense of intercalated cell fates, inactivation of Notch signaling favors the intercalated cell fates [36,37,50]. Within the developing collecting ducts the ligands Jag1 and Dll1 become restricted to the minority intercalated cells which signal to the neighboring cells to activate Notch receptors. Notch receptor activation is hypothesized to mediate a lateral inhibition signal to repress the selection of the minority intercalated cell fate resulting in a “salt and pepper” patterning of principal and intercalated cells much like in the lateral inhibition model of neuronal versus epidermal fate selection in Drosophila [55]. The earliest genes activated by ectopic expression of activated Notch1 within the developing mouse collecting ducts include *Hes1* and *Elf5* [50]. This occurs prior to repression of *Foxi1*, an intercalated cell specific transcription factor. Whereas Hes1 is a known Notch signaling target and is likely to mediate repression of intercalated cell fate selection, *Elf5* is expressed specifically in principal cells and it is required for normal level of expression of principal cell specific genes, such as *Aqp2* [50]. It remains to be determined how *Elf5* is activated in a principal cell specific manner by Notch signaling. One possibility is that Hes1 represses intercalated cell specific transcription factors, similar to pro-neuronal transcription factors in the lateral inhibition model of neuronal cell fate selection. These intercalated cell specific factors are likely to be upstream of Foxi1 and may mediate repression of principal cell specific genes such as *Elf5*. Other intercalated cell factors include the grainyhead family member Tfcp2l1. Interestingly, inactivation of *Tfcp2l1* results in reduced *Jag1* expression and failure of duct cells to select the intercalated cell fate [52].

In addition to patterning cell types within the collecting duct, altering Notch signaling affects ureteric bud branching. Overexpression of *Jag1* (by Hoxb7cre promoter) yields a variety of phenotypes, including hypoplastic kidneys, tubular cysts, and unilateral aplasia [26]. All these phenotypes are likely a consequence of impaired ureteric budding and branching [26]. However, it remains to be determined whether there is an endogenous role for Notch signaling in ureteric budding and branching.

## 3. Notch Signaling in Adult Kidney Maintenance, Repair, and Fibrosis

While the role of Notch signaling in kidney development is well established (as reviewed above), the requirement for ongoing Notch signaling in the adult kidney is only beginning to be unraveled. Knowing the adult kidney functions of Notch signaling is important as therapies that target Notch signaling have been considered for treating various diseases including end stage kidney disease [56]. Notch signaling activity is generally considered to be down-regulated once kidney development is complete [57,58,59]; however, it does not go away completely as recent studies reveal continued Notch signaling in the adult mouse kidney [43,60].

### 3.1. Sustained Activation of Notch Signaling Promotes Chronic Kidney Disease

**Figure 2:**
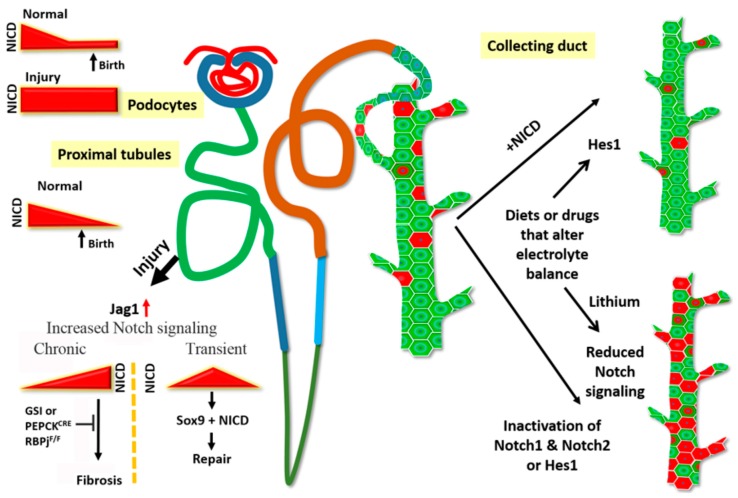
Notch signaling in the adult kidney. Notch signaling is active during nephrogenesis, and the signaling is attenuated after birth in the normal kidney. Reactivation of sustained Notch signaling in the adult kidney either due to genetic activation of Notch signaling or triggered by injury promotes pathological states. Ectopic expression of Notch in mature podocytes results in glomerular dysfunction. The proximal tubules are highly susceptible to injury, and following injury, Jag1 expression increases to activate Notch signaling. Continued high (chronic) Notch signaling leads to fibrosis. This progression can be slowed by blocking Notch signaling either by Gamma secretase inhibitors (GSIs) or by genetic ablation of Notch signaling (PEPCK^CRE^; RBPj^f/f^). However, a transient increase in Notch after injury maybe beneficial as it initiates the repair process mediated by Sox9+ epithelial cells. In the collecting duct, Notch signaling is required to maintain principal cells (green) in their selected fate. Either increase or decrease of Notch signaling perturbs the ratio of principal cells to intercalated cells seen in normal kidney. Diets such as low potassium or drugs such as lithium also increase intercalated cell numbers at the expense of principal cells, and possibly do so by altering Notch signaling activity.

Notch signaling is essential for podocyte development [33], but the signal needs to be attenuated for terminal differentiation of podocytes to occur (Figure 2) [35,38,61]. Waters et al. [47] genetically overexpressed Notch 1 intracellular domain (NICD1) in podocytes to study the role of Notch activation in developing podocytes. At postnatal day (P) 14, mice with overexpressed NICD1 started to demonstrate proteinuria, and histological indications of glomerulosclerosis started appearing at P21. There was a loss of markers of terminal differentiation and re-entry into the cell cycle starting at P21. The deleterious effects of Notch activation were rescued by podocyte specific inactivation of RBPj after the capillary loop stage [47].

Diseases such as focal segmental glomerulosclerosis (FSGS) and diabetic nephropathy are caused by podocyte loss and dysfunction. In human biopsy samples and rodent models of type 1 and type 2 diabetes and of albuminuria and FSGS, Notch signaling is re-activated in the podocytes [62]. This study investigated the reactivation of Notch signaling in a wide spectrum of kidney diseases and reported positive correlation of increased notch signaling with disease severity across the entire spectrum, suggesting elevated Notch signaling as a common mechanism of acquired kidney disease progression. To understand the involvement of Notch signaling in disease progression, Niranjan et al. [57] used a doxycycline-inducible mouse model to express NICD1 in mature podocytes in four-week-old mice. Within a week of doxycycline treatment mice developed massive proteinuria followed by death starting at six weeks of age due to renal failure. Activation of Notch in mature podocytes induced apoptosis possibly by the activation of p53, and the effect of Notch activation was blocked by a podocyte-specific genetic deletion of RBPj, a downstream mediator of Notch signaling. They also found evidence of Notch activation by increasing the levels of Jagged1 by TGFβ.

Additional studies support a role for injury induced increase in Notch signaling in adult kidney tubules promoting tubulointerstitial fibrosis (TIF). Injury induced by either Folic acid injection or unilateral ureteral obstruction (UUO) increased transcripts of some genes of the Notch signaling pathway. Pretreatment of mice with the gamma secretase inhibitor DBZ before initial insult with either folic acid or UUO reduced TIF progression. Further evidence of the involvement or requirement of Notch signaling came from reduction of TIF in tubular epithelial cell-specific knockout of RBPj, and from induction of ICN1 by a doxycycline inducible model that led to TIF. Four-week-old mice administered doxycycline in food for four weeks had an increase in Notch pathway genes and showed signs of fibrosis [56]. Going further into the mechanism, Park et al. [63] used a doxycycline inducible model of overexpressing NICD1 in the tubular epithelium and this led to an increase of principal cells (PCs) and reduction of intercalated cells (ICs). In mouse models of chronic kidney disease induced by folic acid, the typical arrangement of PCs and ICs as seen in normal kidney were lost.

The downstream targets of Notch signaling in promoting kidney fibrosis are largely unknown. Recently, Han et al. [64] reported reduced PGC-1α expression in mice with conditional tubule-specific overexpression of NICD1, as well in models of kidney fibrosis and CKD. Tubules with conditional overexpression of PGC-1α showed reduced effect of Notch-mediated fibrosis. In cultured cells and in vivo, increased NICD1 expression caused defects in mitochondrial structure and biogenesis and led to increased expression of profibrotic, pro-apoptotic genes, features that were reversed with simultaneous overexpression of PGC-1α. Reduced PGC-1α also resulted in lower fatty acid oxidation. Among the Notch pathway genes tested, Hes1 expression showed the highest inverse correlation with PGC-1α expression and chromatin immunoprecipitation identified *Ppargc1a* as a direct target of Hes1. A more recent study [65] identified mitochondrial transcription factor A (*Tfam*) to be both differentially expressed in NICD1-overexpressing kidney tubules and a direct transcriptional target of RBPj. Similar to PGC-1α, Tfam was also down-regulated in NICD1-overexpressing tubules and in multiple models of kidney fibrosis. Tubule-specific knockout of Tfam led to altered mitochondrial structure and increased expression of profibrotic factors. In cultured renal epithelial cells, higher Notch signaling led to decrease in fatty acid oxidation, and this effect could be reversed by increasing Tfam levels. Taken together, the findings of these two studies indicate a role of Notch in perturbing metabolic pathways associated with cellular energy production to promote fibrosis.

### 3.2. Opposing Roles for Notch Signaling Following Kidney Injury

Acute kidney injury (AKI) is triggered by a brief duration of injury that rapidly, within 1–2 days, results in loss of kidney function and decrease of urine output. AKI can be induced by a host of factors, including but not limited to surgery-related injuries, and other physiological events such trauma, stress, infection, and drug toxicity [66]. One in five incidences of AKI result in death, and in those that survive, AKI usually progresses to chronic kidney disease (CKD) [67,68]. While the adult kidney does not have the capacity to form new nephrons, since NPCs are depleted prior to birth in humans or soon after birth in rodents, it does have the capacity for tubular epithelial repair. Animal models developed to study the response to AKI include ischemia-reperfusion injury (IRI) and nephrotoxicity induced by drugs such as cisplatin and folic acid, among others as reviewed previously [69,70,71] have revealed the reactivation of developmentally important signaling pathways such as Notch following epithelial injury. The first report tying the Notch signaling pathway to progression of kidney fibrosis came in 2002, in which a tissue microarray identified several genes of the Notch pathway (notably not Notch2) to be upregulated following the addition of the pro-fibrotic factor transforming growth factor β1 to cultured renal cortical epithelial cells [72]. TGFβ1 is known to be involved in UUO-mediated kidney fibrosis in rats [73]. Among the Notch pathway genes, Jagged-1 expression was elevated the most. Jag1 mRNA and protein levels were also increased in UUO models in mice and when TGFβ1 was injected intraperitonially. Kobayashi et al. [74] using an ischemia-reperfusion injury (IRI) model in rats observed increased Notch2, Dll1, and Hes1 transcript and protein levels following AKI. Immunohistochemistry revealed the co-localization of the upregulated proteins in the proximal tubule S3 segment, which is most susceptible to IRI mediated injury [75]. Using the same rat model of ischemia-reperfusion, Gupta et al. [76] demonstrated a faster recovery after injury and higher cell proliferation rate in the rats pretreated with recombinant DLL4. However, this study did not address the change in Notch signaling downstream of DLL4. In a later study, treatment of proximal tubular epithelial cells resulted in increased expression of Hes1. In a similar model as used by Gupta et al. but with additional right nephrectomy, pretreatment with DAPT reduced the level of Notch reactivation with simultaneous decrease of inflammation and apoptosis [77]. In this study, pro-inflammatory and pro-apoptotic factors first increased at 24 h followed by a decrease at 48 and 72 h. From the studies of Chen et al. [78] it appears that there is an initial increase in apoptotic factors immediately after injury, after which Notch signaling acts through the STAT3 pathway to increase the anti-apoptotic factor Survivin, possibly as a feedback mechanism to reduce the duration of apoptosis. DAPT pretreatment before IRI therefore reduces this duration further to protect against AKI. Increase in Notch pathway genes Jag1, Notch1, and HeyL was also demonstrated in Folic acid-induced kidney injury and in CKD patient samples [56].

While many studies suggest a pathological role for increased Notch signaling following AKI as discussed above, recent studies reveal beneficial roles for Notch signaling activation following AKI. Following AKI-induced tubular epithelial damage, repair mechanisms are initiated to restore nephron structure and function. The repair is mediated by the neighboring less damaged tubular epithelial cells [79], but the key players of this repair response have not been identified. TRAP (translating ribosome affinity purification) identified Sox9 to be actively translated in the nephron tubules in an early (4 h) response to IRI [80]. Normally, Sox9+ cells are rarely found in uninjured epithelium. Following injury, however, Sox9 was activated before other known biomarkers for kidney injury. Moreover, expression of these biomarkers in the tubules remained elevated 28 d post-injury. Interestingly, even though these rare Sox9+ cells contribute to repair, their small numbers are probably not enough for the injury response, as lineage tracing studies show the injured tubules actually activate *de novo* Sox9+ expression in response to injury. The Susztak group showed that these highly proliferative Sox9+ cells do not express any transcription factors commonly seen in the developing kidney, such as Lgr5, Pax2, and Six2, suggesting their progenitor nature. In vivo lineage tracing showed these Sox9-positive cells could give rise to all epithelia of the kidney except collecting duct and glomeruli. This cell population showed increased proliferation in the adult kidney after injury by either folic acid or IRI. Eventually, these cells led to tubule generation in different segments of the kidney [81]. The expression of Notch pathway genes in kidney side population cells [82] and involvement of Notch in self-renewal and cell fate commitment of stem cells [83] make Notch a high priority candidate in kidney regeneration. Indeed, these Sox9-positive cells showed high level of Notch signaling and overexpression of NICD1 in the Sox9-positive population showed improved renal histology [81]. More recently, in a model of left kidney partial nephrectomy, the Notch inhibitor DAPT was found to abolish the increase of Sox9-positive cells, showing Notch to be a key player in regulating epithelial repair in kidney regeneration after injury (Figure 2) [84].

### 3.3. Notch Signaling in the Maintenance of Mature Kidney Cell Types and Remodeling of Epithelial Segments

The collecting duct segments of the kidney consist of two broad cell types, the principal cells (PCs) and the intercalated cells (ICs) intermingled with each other. These two cell types arise from a common progenitor, and proper functioning of the collecting duct system requires the relative ratio of these cell types be maintained throughout the tubule. These cell types are characterized by the presence of specific marker proteins. Differentiation of the ICs require the presence of the forkhead transcription factor Foxi1 [85], and several groups have shown the essential role of Notch signaling in determining the PC fate during development [36,37,50]. Lithium, a drug used in the treatment of bipolar disorder often leads to a urine concentrating defect (acquired NDI) [86], possibly due to alteration of the ratio of PCs to ICs in adult humans, and in rodent models of lithium-induced NDI, withdrawal of the drug restores the normal ratio of PCs to ICs in the collecting duct. This indicates a plastic nature of the cells even after they have achieved terminal differentiation. This observation was key to the hypothesis that signaling mechanisms involved in cell fate decisions need to be sustained beyond development to maintain cells in the fate they have selected. Since Notch signaling is one of the essential pathways to determine the PC fate, to test this hypothesis, Mukherjee et al. [60] inactivated Notch signaling in the tubule epithelium and collected duct in adult mice using a doxycycline-inducible model and observed a decrease in the ratio of PC to IC (Figure 2). Furthermore, lineage tracing experiments proved conclusively that inactivation of Hes1 (and hence Notch signaling) in PCs caused the PCs to convert to ICs. Additionally, soon after inactivation of Hes1, intermediate state cells expressing markers of both PCs and ICs, were detected [60]. Without inactivation of Notch signaling, lineage tracing of Elf5-expressing PCs revealed that a small percentage (0.2%) of labeled-PCs convert to ICs over a five-week time period [60]. Consistent with this, Park et al. have determined by single-cell-RNA-sequencing of cells isolated from whole adult mouse kidneys that there are cells in between a PC state and an IC state expressing markers of both cell types. Park et al. however claim that it is the ICs that are converting to PCs, and that this conversion increases in disease conditions in which Notch is activated. In support of this idea they observed that ectopic overexpression of NICD1 in kidney tubular epithelial cells, including in ICs, using the Pax8->rtTA doxycycline inducible system led to an increase of PC numbers when compared to wild-type and a depletion of ICs [63]. Taken together, these observations strongly point to a requirement for continued Notch signaling in the adult kidney epithelium to maintain the functional integrity of differentiated cells.

Other studies point to a role for increased Notch signaling activity in the epithelial cells of the distal nephron segments to remodel the cell types in response to inactivation of the sodium chloride cotransporter (NCC). The distal nephron remodeling process is observed to involve induction of Jagged1/NOTCH signaling, and increased Hes1 levels. This then results in an increase in PCs within the cortical connecting tubule and replacement of acid-secreting alpha-ICs with pendrin expressing-ICs [43]. Hence, modulating Notch signaling within the adult kidney epithelial cell types of the distal nephron and collecting duct segments may be part of chronic physiologic adaptive mechanisms.

Several developmentally important signaling pathways, such as Wnt signaling and hedgehog signaling are re-activated after injury (reviewed in [87]). Similarly, numerous studies have reported the activation of Notch in response to both acute and chronic renal injury. However, continued activation of Notch signaling promotes renal fibrosis (Figure 2) in chronic kidney diseases such as diabetic nephropathy. These two statements underline the conundrum currently existing surrounding the role of Notch in kidney injury: Is Notch reactivation in adult the cause or result of kidney pathology? Based on current literature, it is possible to speculate a scenario where Notch signaling occurs at low levels post-development to maintain mature cell types allowing for normal renal physiology. Injury to the kidney reactivates the Notch signaling, which leads to cellular de-differentiation, regeneration, and repair (Figure 2). However, prolonged activation, which may be caused by the absence of cellular machinery normally present during development to shut off Notch signaling at a specific time, is detrimental to kidney health, leading to the pathological state. The Wilms’ tumor suppressor gene WT1 is essential for podocyte development, maintenance and function, and mutations in WT1 are associated with FSGS. WT1 continues to be expressed in the mature podocytes, and deletion of WT1 in mature podocytes by a tamoxifen-inducible system led to podocyte loss, increased Notch signaling and glomerulosclerosis. Based on this study, disease onset begins at four days following initiation of WT1 deletion, and by day six extensive glomerulosclerosis occurs. By this time, several members of the Notch signaling pathway show up-regulated transcript levels, suggesting disease onset and Notch activation happens at about the same time [88]. In a study by Sorensen-Zender et al. [89], aged mouse kidneys sustained the reactivated Notch signaling after IRI much longer than younger mice, and the prolonged Notch activation hampered the repair process and led to a senescent and pro-fibrotic condition. This might suggest younger tubules have a higher ability to regulate the period of Notch activation. Taken together, current data suggests a very fine-tuned temporal regulation of Notch activation after injury, the mechanism of which is still largely unknown.

## 4. Human Kidney Diseases Associated with Altered Notch Signaling

Consistent with roles for Notch signaling in kidney development and maintenance, genetic mutations either inherited or acquired *de novo* in genes coding for Notch signaling pathway components are associated with renal diseases (Table 2 and Table 3), including Alagille syndrome (ALGS), Hajdu-Cheney syndrome (HCS), and congenital anomalies of the kidney and urinary tract (CAKUT). Additionally, diabetic nephropathy and renal carcinoma are associated with altered expression levels of Notch signaling components in humans.

### 4.1. Alagille Syndrome (ALGS)

Alagille syndrome (ALGS; MIM118450) is an autosomal dominant disorder that can affect multiple organs of the body including the liver, heart, skeleton, eyes, and kidneys. ALGS is caused by heterozygous mutations in *JAG1* or *NOTCH2* genes that are crucial components of Notch signaling pathway. Analysis of missense mutations in cell-based assays and mouse models indicate that the disease occurs due to loss-of-function [100,101]. However, it remains possible that some missense mutations behave in a dominant negative manner. The frequency of this disease was estimated to be 1 in 70,000 based on the clinical observation of the symptoms associated the disease [102]. Due to the high variability in these symptoms, some mildly affected or clinically unaffected carriers were missed in the symptoms based diagnosis estimation [94]. Accordingly, ALGS diagnosis based on genetic testing has increased the estimated incidence of ALGS to 1 in 30,000 [103]. To date 694 pathogenic variants for *JAG1* and 19 patients with pathogenic variants in *NOTCH2* have been described [104]. The majority of the patients (94.3%) have mutations in *JAG1* [90] and another 2.5% of patients have mutations in *NOTCH2* gene [90,91]. The remaining 3.2% of patients confirmed with ALGS-associated symptoms do not have mutations in *JAG1* or *NOTCH2* genes. Based on mouse studies which implicate the canonical Notch signaling pathway in kidney disease the genetic basis in the remaining 3.2% of patients could be in additional Notch signaling genes. Often phenotypic findings are highly variable in terms of severity and clinical significance even within the same family members although they possess similar mutations. Mutations in modifier genes and or environmental factors may be causing this variability.

Renal anomalies are found in around 40% of the ALGS patients with mutations in *JAG1*. The ALGS associated renal anomalies include multicystic dysplastic kidneys, renal tubular acidosis, vesicoureteral reflux, obstruction, chronic renal failure, end-stage renal disease, acute kidney injury, renal lipidosis, renal artery stenosis, focal segmental glomerulosclerosis, duplex collecting system, and other renal diseases in a minor proportion [91,94]. Currently there is no cure for the Alagille syndrome, and treatment options include organ transplantation in extreme cases [91], although risk of rejection and immunosuppression is a risk. An attractive future therapy may include treating with small molecules that boost Notch signaling only when and where Notch signaling normally occurs.

### 4.2. Hajdu-Cheney Syndrome (HCS)

Hajdu-Cheney syndrome (HCS, MIM102500) is a rare genetic disorder with prominent features including bone abnormalities and to a lesser extent renal and cardiac abnormalities among others [105]. The disease was first described by Hajdu in 1948, later was reported further by Cheney in 1965. HCS is caused by mutations in exon 34 of *NOTCH2* gene, that results in the truncation of NOTCH2 protein and the absence of the c-terminal pest domain, which contains sequences necessary for the ubiquitinylation and degradation of NOTCH2 following association of the intracellular domain with RBPJ and Mastermind. The absence of the PEST domain is predicted to increase the half-life of NOTCH2 resulting in gain-of-function effects [106]. So far there are less than 100 HSC cases reported in the medical literature [106,107], and HSC disease frequency is likely to be low due to the fact that only mutations that occur in exon 34 of NOTCH2 are correlated with HSC. Renal abnormalities occur in around 10%–18% of HSC patients, but maybe underestimated as ultrasound technology was not available for diagnosis renal abnormalities when many cases were diagnosed. Various renal anomalies observed in the HCS patients include cystic kidneys, hypoplasia, vesicoureteral reflux, glomerulonephritis, hypertension, and chronic renal failure [96,107]. Similar to ALGS, HCS also exhibits high variability in symptoms and severity even within the family members. In a particular case report, a mother had HCS syndrome with no renal anomalies while her infant with HCS syndrome had multiple bilateral small renal cortical cysts [108]. Brennan and Pauli studied 57 cases of HCS, and reported that 10 HCS patients (18%) had some form of kidney disease with cystic kidneys being the most common abnormality, occurring in 14% of the patients [107]. In 70% of HSC patients with kidney disease, the renal anomalies were identified before or during early childhood. Mutations in NOTCH2 exon 34 have also been linked to a disease termed Serpentine fibula polycystic kidney syndrome (SFPKS, MIM600330), and is likely to be part of the spectrum of phenotypic variability seen in HCS [109]. Considering the overlap in the genetic basis as well as in the disease symptoms between HCS and SFPKS both HCS and SFPKS are referring to the same disease [110].

### 4.3. Congenital Anomalies of the Kidney and Urinary Tract (CAKUT)

Congenital anomalies of the kidney and urinary tract (CAKUT) includes a spectrum of anomalies affecting the kidney, the urinary tract, or both [111]. CAKUT occurs in 0.3% to 0.6% of live births and accounts for 34%–59% of chronic kidney disease (CKD) and for 31% of all cases of end-stage kidney disease (ESKD) in children in the United States. Thus far, mutations in 185 genes are linked to CAKUT in mice, 179 genes are linked with syndromic CAKUT in humans and mutations in 40 monogenic genes for isolated CAKUT are known [112]. Considering that Notch signaling is involved in many aspects of kidney development it should not be surprising that mutations in *JAG1* or *NOTCH2* are associated with CAKUT. What is perhaps surprising is that mutations in other Notch pathways genes have so far not been associated with CAKUT. However, the genetic basis of CAKUT in most patients remains to be determined.

### 4.4. Diabetic Nephropathy

Diabetic nephropathy is also called diabetic kidney disease (DKD), and is the leading cause of end-stage kidney disease and occurs in approximately 40% of patients who are diabetic. Diabetic nephropathy occurs due to hyperglycemia observed in diabetic patients, although the symptoms are quite variable between type I versus type II diabetes [113]. Among the several factors mediating the development of the diabetic nephropathy, Notch signaling appears to play an important role. Increased renal Notch signaling is detected in the patients with diabetic nephropathy, as evidenced by increased expression of cleaved Notch1 [62]. To identify genetic contribution for DKD, genome wide association study was conducted involving 40,340 subjects with type I or type II diabetes among them 18,582 subjects had DKD. In spite of having a large set of subjects, a clear genetic correlation with kidney disease was not made. Another study examining the genome-wide cytosine methylation pattern of tubular epithelial cells micro-dissected from human kidneys of control and DKD subjects identified changes in the methylation levels predictive of altered gene expression levels of important renal transcription factors [114]. Exact mechanism for upregulation of Notch pathway is unknown at this time. It is also suggested that, diabetic nephropathy may originate from metabolic dysregulation, which can alter various signaling pathways including Notch signaling pathway. The increased expression of Notch signaling components Notch1, Notch2, and Jagged1 in the kidneys of diseased patient samples, in animal models and cell culture experiments [62,115] has led to the hypothesis that reducing the level of Notch signaling could help in preventing or delaying the disease severity. Inhibition of Notch1 signaling through DAPT or specific shRNA knockdown strategies, significantly abrogated VEGF activation and nephrin repression in high glucose stressed cells and reduced proteinuria in diabetic rats [115]. In another study by Rojas et al. [116] treatment with Gliquidone significantly reduced expression levels of Notch signaling components Notch1, Jag1, and Hes1 in a dose dependent manner and delayed the progression of diabetic nephropathy in mouse models. These studies collectively suggest increased levels of Notch signaling is one the mediator of diabetic nephropathy, and restoring Notch signaling levels through pharmacological compounds or gene therapy may delay the onset of diabetic nephropathy.

### 4.5. Kidney Cancers

Renal carcinoma is one of the most commonly occurring cancers with nearly 270,000 cases diagnosed yearly and accounting for 116,000 deaths annually [117]. Notch signaling pathway is associated with both oncogenic and tumor suppressive roles and has been documented in various cancer types including renal carcinoma. Among Notch signaling components NOTCH1 plays a critical role. NOTCH1 expression is significantly elevated in metastatic tumors in T1 stage, with tumor size positively correlating with the elevated expression levels and the average size of metastatic tumors being significantly larger non-metastatic tumors in T1 stage of clear cell renal cell carcinoma [118]. This study by itself is suggestive of an oncogenic role for Notch signaling in kidney cancers.

Renal cell carcinoma can be further divided into at least three subtypes: Clear cell renal cell carcinoma, papillary renal cell carcinoma, and chromophobe renal cell carcinoma. Some of Notch signaling components are uniquely associated with each of these subtypes based on mRNASeq data of the patient samples [119]. Low expression of ADAM10 and HES1 are observed in clear cell renal cell carcinoma, while lower expression of HES5 and JAG1 are observed in papillary cell renal cell carcinoma and lower expression of NOTCH2 is observed in chromophobe renal cell carcinoma. Additionally, the expression of FHL1B/KyoT3, an inhibitor of Notch signaling mediated transcription, is elevated in human class 1 papillary renal cell carcinoma [42]. Taken together with the observation that conditional Notch2 inactivation in the mouse kidney results in renal tubular cysts with microadenomas that could be a precursor to papillary renal cell carcinoma, Notch signaling may play a tumor suppressor role in the kidney [42]. Hence, both increased and decreased levels of Notch signaling have been correlated with kidney cancers. Animal models of Notch dependent kidney cancers will be necessary to prove the ability of altered levels of Notch signaling to cause renal carcinoma.

## 5. Conclusions

Mouse studies have revealed many functions for Notch signaling in kidney development, in the maintenance of adult kidney cell types and in the response to kidney injury. The myriad functions of Notch signaling may in part explain the high degree of variability in the onset and severity of kidney disease observed among individuals with the same genetic defect in the Notch signaling pathway. ALGS patients with the same *NOTCH2* mutation may have no renal defects, or congenital cystic kidney disease or late adult onset kidney disease. This is similar to mouse lines with a sensitized level of Notch signaling during kidney development some of which develop small kidneys while others develop normal sized kidneys. The sensitized level of Notch signaling likely allows for normally insignificant genetic variations, or dietary changes, or environmental factors to affect the severity of the kidney disease. For example, normally a null mutation in one allele of *Notch1* does not cause kidney disease in mice, however when we combine this sensitized level of Notch signaling with the conditional inactivation of *Notch2* in the developing kidney nephron progenitors this results in development of multicystic hypoplastic kidneys. These mouse studies suggest that additional variations in the Notch signaling pathway genes could be present in some ALGS patients with severe kidney disease in addition to a mutation in *JAG1* or *NOTCH2*. Additional genetic modifiers may also be present in genes involved in unique functions that Notch signaling mediates in the kidney of ALGS patients with severe kidney disease. The role of Notch signaling in the maintenance of adult kidney cell types, or in the distal tubule cell type remodeling to adapt to chronic dietary changes, or in renal epithelial repair following injury is suggestive that a secondary event in adults with reduced Notch signaling could trigger late onset of kidney disease in some ALGS patients.

## Figures and Tables

**Table 1:** Phenotypes of mouse models with Notch signaling activity modulated within the developing kidney.

Regulatory Region Driving Cre/Time Point and Place in Renal System Where Inactivation Occurs	Mouse Model: Genetically Modified Gene and Observed Phenotype	Reference
Hypomorphic Notch2 alleles (Notch2 ^del1/del1^)	Notch2 ^del1/del1^: Perinatal lethality with hypoplastic kidneys, vascular lesions near cortical region, defective glomerulogenesis, and lack of proper glomeruli	[32]
	Notch 2 +/del1, Jag1 +/−: Half sized kidneys, decrease in glomeruli number, defective glomeruli	
	Notch 2 +/del1, Dll1 +/−: No kidney defects	
Global deletions and global overexpression	Psen1 −/−, Psen2 −/− with rescue of severe pre-natal lethality by PSEN1 human expression: Lethality at 2 h post birth, smaller kidneys that lack comma and S-shaped bodies, glomeruli, proximal tubules, and distal nephron tubule begins formation but does not fully mature	[34]
Hoxb7->Cre/collecting duct	Human Jag1 gene overexpression: Variable phenotypes including cysts, decreased nephrons, hypoplastic kidneys, hydropelvises, hydroureters along with drastically lowered GDNF expression levels	[26]
Pax3->Cre/Metanephrogenic Mesenchyme Pre-11.5	Notch2 f/f: Lethality between P1 and P2 due to renal failure with no filtration apparatus present, smaller kidneys with collapsed renal pelvis, no proximal tubules with intact distal tubules, and lack of proximal podocytes	[40]
Pax2-Cre/Pre-10.5 renal development	Notch2 f/f: No podocytes or proximal tubules present in kidneys of mutants	
	RBPJ f/f: Death by E13.5, explant culture revealed lack of proximal tubule and podocytes Notch1f/f: Explant kidney shows no phenotype	
Six2->GFP-Cre/E12.5 onwards in cap mesenchyme	NICD overexpression: Hypoplastic kidneys with only one ureteric branch	
Neph->Cre/Podocytes only	NICD overexpression: Proteinuria caused by impaired glomerular filtration selective permeability with progressive glomerulosclerosis and a decrease in mature marker expression (Wt1, Nphs1, and Nphs2) with increased cell cycle activity and increased Pax2 expression	[47]
Podocin->Cre/Podocytes only	RBPJ f/−: No observable phenotype	
	RBPJ f/−, NICD overexpression: Rescue of severe selective filtration defect from increased Notch and rescue of Glomerulosclerosis	
Hoxb7->Cre/collecting duct	Mib1 f/f: Unilateral or bilateral hydronephrosis of distended kidneys at P17, reduced number of principal cells, and increased number of intercalated cells	[36]
Six2->GFP-Cre/E12.5 onwards in cap mesenchyme surrounding ureteric bud tips	N2-ICD overexpression: Lethality after birth and kidneys have glomerular cysts, dilated renal tubules, and thin cortexes	[45]
Six2->GFP-Cre/E12.5 onwards in cap mesenchyme surrounding ureteric bud tips	Notch2 f/f: Low percentage of renal cysts at P0 with a formation of micro adenomas (proliferating cells) by 52 weeks of age	[48]
	Notch1 f/f: 30% of mutant mice have renal cysts at P0	
	RBPJ f/f: Lethality by P2 in mutants, kidneys have few glomeruli and proximal tubules	
Rarb2->Cre/Condensing mesenchyme	RBPJ f/f: Large proximal tubule cysts present in mutant kidneys	
Six2->GFP-Cre/E12.5 onwards in cap mesenchyme surrounding ureteric bud tips	Notch2 f/f: 31% of mutants have smaller kidneys with fewer glomeruli	[42]
	Notch1 +/f; Notch2f/f: 67% of mutants have smaller kidneys with fewer glomeruli, increased blood urea nitrogen levels at birth, reduced life span	
	Notch1 f/f, Notch2 f/f: Lethality at P1 with compromised renal function (blood urea nitrogen), few proximal tubules with very few glomeruli	
	RBPJ f/f: Mutants mice die at P2 due to insufficient filtration in small kidneys Kidneys have few mature nephrons and no S-Shaped body during development with few proximal tubules	
Pax3->Cre/Metanephric Mesenchyme Pre-11.5	Notch2 f/f, Mint f/f: Mint inactivation partially rescues Notch2-deficient phenotype by increasing the number of proximal nephron segments forms	
Rarb2->Cre/Condensing mesenchyme	RBPJ f/deletion: Hypoplastic kidneys that develop cysts with death due to increased blood urea levels causing renal failure Fewer proximal tubules formed, were dilated and cyst-like, with few glomeruli that were functioning Note: Some progenitors escape RBPJ inactivation leading to the development of the present proximal tubules	[49]
Hoxb7->Cre/Collecting duct	Adam10 f/f: Hydronephrosis in 30% of mutants with increased water intake, increase urine output, and decreased osmolality; decrease in principal cells and an increase in intercalated cells in collecting duct	[37]
Six2->GFP-Cre/ E12.5 onwards in cap mesenchyme surrounding ureteric bud tips	Six2-3XFlag overexpression: Decreased differentiation from mesenchymal progenitors to epithelial tubules	[25]
	RBPJ f/f: Increased Six2+ cells found deeper in medullary, decreased nephron endowment	
	N1 f/f, N2 f/f: Increased Six2+ cells found deeper in medullary, decrease in the number nephrons, lack of development of proper nephrons, and renal vesicles failed to form S-shaped bodies Important note: Mosaicism in Cre positive cells formed some nephrons	
Hoxb7-Cre/Collecting duct	Rbpj f/−: Increased intercalated cells in collecting duct, decreased expression of Elf5	[50]
	PS1 −/f and Ps2 −/−: Increased intercalated cell gene expression with decreased principal cell gene expression	
	Rosa +/NICD: Increased principal cell gene expression including Elf5	
Cdh16->Cre/Collecting duct and connecting tubule	Elf5 F/del: Slight decrease in principal cell gene expression	
Wnt4->GFP-Cre/Pre-tubular aggregates	Notch1 f/f, Notch2 f/f: Lack of developed nephrons and not just proximal tubules as previously noted, no premature depletion of mesenchymal nephron progenitors	[51]
	NICD overexpression: No effect on nephron differentiation, glomerulocystic kidney phenotype	
Six2->GFP-Cre/E12.5 onwards in cap mesenchyme surrounding ureteric bud tips	Rosa LacZ/NICD: Glomerulocystic kidneys within mutants; heterogeneous nephron cell population segmenting in the nephron, and not just proximal tubule population as previously found	
Cdh16->Cre/Collecting duct and connecting tubule	Jag1 f/f: Increase in collecting duct cell types expressing both principal and intercalated proteins with tubules enlarged as well as enclosing fragments of the tubule; hydronephrosis present in adult mice	[52]
	Tfcp2l1 f/f: Absence of intercalated cell development in collecting ducts	
Atp6v1b1->Cre/Intercalated cells of the collecting duct (possibly leaky expression in principal cells)	Jag1 f/f: Increase in cells expressing both principal and intercalated proteins within the collecting duct with decreased principal cells	
	Tfcp2l1 f/f: Standard ratio of principal to intercalated cells in collecting duct at two weeks of age; by two months the collecting duct contains half of the intercalated cells with decreased intercalated protein expression when compared to wild-type	
EllA-Cre/ Early embryogenesis; one cell zygote stage	Tfcp2l1 f/f: Lethality post birth; elimination of intercalated cells in collecting duct	

**Table 2:** Renal anomalies in humans associated with mutations in *NOTCH2.*

Human Disease	Genetic Mutation	Kidney Defect	References
Alagille Syndrome	*NOTCH2 p.Cys444Tyr (C444Y)/ECD*	Small congenital cystic kidney disease	[90]
	*NOTCH2 c.5930−1G→A/ICD*	Tubular acidosis and dysplastic kidneys	[90]
	*NOTCH2 p.Cys373Arg/ECD*	Vesico-ureteric reflux	[91]
	*NOTCH2 p.Arg2003X/ICD*	Echogenicity of kidneys	[91]
CAKUT	*NOTCH2 p.Tyr1186Asn/ECD*	Vesicoureteral reflux	[92]
	*NOTCH2 p.Arg2256His (R2256H)/ICD*	Small dysplastic kidney, ureterovesical junction obstruction	[92]
	*NOTCH2 p.Arg2298Trp/ICD*	Hydronephrosis	[92]
Hajdu–Cheney syndrome	*NOTCH2 (Gln2389X)/ICD*	Polycystic kidneys	[93]

**Table 3:** Kidney diseases associated with alterations in Notch receptors and ligands.

Gene	Disease	Kidney Disease	References
*JAG1*	ALGS	Dysplasia (generalized, focal, with vesicoureteral reflux, with renal insufficiency), renal tubular acidosis, vesicoureteral reflux, hydronephrosis, obstruction (retero-pelvic junction, with hydronephrosis), chronic renal failure, endstage renal disease, acute kidney injury, renal lipidosis, renal artery stenosis (bilateral), focal segmental glomerulosclerosis, duplex collecting system	[94,95]
*NOTCH2*	ALGS	Severe infantile renal disease (small kidneys with cysts bilaterally, renal tubular acidosis, and renal insufficiency), proteinuria that resulted in renal failure, tubular acidosis and dysplastic kidneys, vesicoureteral reflux, echogenicity of kidneys, Neonatal renal failure	[90,91]
*NOTCH2*	HCS/SFPKS	Cystic disease, hypoplasia, vesicoureteral reflux, glomerulonephritis, hypertension, chronic renal failure, bilateral dysplastic kidneys with numerous, small parenchymal cysts, associated with bilateral, high-grade vesicoureteral reflux	[96,97]
*NOTCH3*	CADASIL	Focal segmental mesangial proliferation, the loss and degeneration of arterial medial smooth muscle cells and arterial intimal thickening. Immunofluorescence analysis of glomeruli revealed IgA deposition in the mesangial area. Electron microscope analysis revealed electron-dense deposition also in the mesangial area. In addition, granular osmophilic material (GOM) was observed in the extra-glomerular mesangial area and around the vascular smooth muscle cells	[98]
*NOTCH3*	CADASIL	Chronic kidney disease, renal histological analysis showed severe arteriosclerosis and mild interstitial fibrosis	[99]
*NOTCH1*	DKD	Higher Notch1 expression observed in glomerulosclerosis	[62]
